# Association of education with occurrence of delirium in patients from an emergency department

**DOI:** 10.1590/S1980-5764-2016DN1003005

**Published:** 2016

**Authors:** Simone Sieben da Mota, Vera Beatriz Delgado, Artur Francisco Schumacher-Schuh, Marcia Lorena Fagundes Chaves

**Affiliations:** 1 NA, MSc, Programa de Pós-Graduação em Medicina: Ciências Médicas, Faculdade de Medicina, Universidade Federal do Rio Grande do Sul (UFRGS), Porto Alegre RS, Brasil.; 2 NA, PhD, Serviço de Enfermagem Psiquiátrica, Hospital de Clínicas de Porto Alegre (HCPA), Porto Alegre RS, Brasil.; 3 MD, PhD, Ambulatório de Demência, Serviço de Neurologia, HCPA.; 4 Programa de Pós-Graduação em Medicina: Ciências Médicas, Faculdade de Medicina, Universidade Federal do Rio Grande do Sul (UFRGS), Porto Alegre RS, Brasil. Ambulatório de Demência, Serviço de Neurologia, HCPA. Departamento de Medicina Interna, Faculdade de Medicina, UFRGS.

**Keywords:** delirium, education, emergency

## Abstract

**Background:**

Delirium is a neuropsychiatric syndrome with multiple etiological factors. Evaluation of delirium in different settings, especially the Emergency Department (ED) pertaining to different regions of the world with patients from different cultural and educational backgrounds is needed.

**Objective:**

To determine the prevalence of delirium and its association with education in an ED in Brazil during a 6-month period. Methods: Patients aged >18 years were randomly selected from ED admissions. The instruments Confusion Assessment

**Method:**

(CAM) scale, Mini-Mental State Examination (MMSE), Wechsler Logical Memory (WLM) and Charlson comorbidity score were applied to evaluate delirium, cognitive status, and comorbidities.

**Results:**

The prevalence of delirium was10.7%. Delirium patients had significantly lower education, MMSE and WLM (immediate and delayed) scores, with 97.4% presenting episodic memory impairment. Patients with delirium had more history of neurological disorders. Three logistic regression models evaluating the association of variables with delirium were developed. Age and MMSE were retained in the first model, WLM scores in the second, and education in the third.

**Conclusion:**

To the best of our knowledge, this is the first study estimating the prevalence of delirium in a Brazilian ED. Lower education was associated with the occurrence of delirium.

## RESUMO

**Introdução:**

*Delirium* é uma síndrome neuropsiquiátrica, com fatores etiológicos múltiplos. A avaliação de *delirium* em diferentes ambientes, especialmente no Serviço de Emergência (SE) de diferentes regiões do mundo e com diferentes características culturais e educacionais é necessária.

**Objetivo:**

Determinar a prevalência de *delirium* e associação com educação em um SE no Brasil, durante seis meses.

**Métodos:**

Foram randomizados aleatoriamente no SE os pacientes com idade acima de 18 anos. *Escala Confusion Assessment Method* (CAM), Mini Exame do Estado Mental (MEEM), o teste de Memória Lógica de Wechsler (MLW), e o escore de comorbidade de Charlson foram aplicados para avaliar *delirium*, status cognitivo, e comorbidades.

**Resultados:**

A prevalência de *delirium* foi 10,7%. Os pacientes com *delirium* apresentaram significativamente menor escolaridade, escores mais baixos no MEEM e MLW (imediato e tardio), sendo que 97,4% apresentava comprometimento de memória episódica. Pacientes com delirium apresentaram mais história de transtorno neurológico prévio. Três modelos de regressão logística para delirium foram realizados. No primeiro, idade e MEEM foram mantidos no modelo final. No segundo, MLW imediato e tardio; e no terceiro, apenas educação.

**Conclusão:**

Este é o primeiro estudo brasileiro, de acordo com nosso melhor conhecimento, a estimar a prevalência de *delirium* em serviço de emergência. Nível educacional mais baixo foi associado com ocorrência de *delirium*.

## INTRODUCTION

Delirium is a neuropsychiatric syndrome with multiple etiological factors, characterized by cognitive dysfunction with acute onset, especially attention. It is usually associated with an underlying general medical condition.^1^ This syndrome is associated with increased morbidity and mortality, persistent functional and cognitive decline, longer hospital stay, higher rates of nursing home placement and increased health care costs.^2^ This disorder remains a poorly understood condition and is frequently unrecognized by health care professionals, despite its clinical importance and economic impact.^3^

Delirium is more common in older individuals (>60 years),^4^ ^,^ ^5^ but can occur at any age.^6^ The frequency of delirium is highly variable. Prevalence in the general population according to age group is 0.4% for persons >18 years, 1.1% for those >55 years, and 13.6% for elderly individuals >85 years old.^7^ In a recent systematic review on the occurrence and detection of delirium within the emergency care setting, frequency of delirium at admission to the ED ranged from 7% to 20%.^8^

Delirium susceptibility varies between individuals. Delirium is usually the manifestation of a complex interaction of a vulnerable patient exposed to harmful insults or precipitating factors. Previous studies have clarified the vulnerability factors for delirium including frailty, cognitive impairment, vision or hearing disability, and comorbidity.^9^ ^,^ ^10^ The concept of cognitive and brain reserve represents important new models to capture this vulnerability to delirium. Cognitive reserve has not been widely studied in the context of delirium. A secondary analysis of two large hospital-based prospective cohorts involving older adults (≥70 years) examined the role of educational attainment and of risk for delirium in patients who were delirium-free at admission.^11^ Based on educational difference between the groups, a positive five-year difference in educational attainment was associated with a 1.6- fold decrease in the odds of delirium. The authors concluded education was strongly associated with the risk of delirium. However, the majority of information on frequency of delirium in ED is derived from developed countries, where educational attainment, as an estimate of cognitive reserve, is higher. Therefore, further evaluation in different settings, especially within ED pertaining to different regions of the world involving patients from different cultural and educational backgrounds is needed. The objective of the present study was to evaluate the prevalence of delirium and its association with education in an Emergency Department of a large university hospital in Brazil using a standardized instrument (Confusion Assessment Method - CAM), controlling for age and underlying disorders. 

## METHODS

A cross-sectional investigation was carried out during different dayshifts and week days within the ED of the Hospital de Clínicas de Porto Alegre between March and August 2013. The study was approved by the local Ethics Committee (#11-0559), and all participants and/or a proxy signed the consent form. 

Participants and procedures. Patients aged >18 years were randomly selected from daily ED admissions. Individuals with language barrier, severe aphasia, intubation, coma (Glasgow scale <11), respiratory isolation and those critically-ill or clinically unstable were excluded (SOFA score >8).

Delirium was assessed by trained research nurses from Monday to Friday during different dayshifts (morning, afternoon, and evening) using the CAM,^12^ ^,^ ^13^ Mini-Mental State Examination (MMSE)^14^ and Wechsler's Logical Memory (WLM) test from the Wechsler Memory Scale - III.^15^ ^,^ ^16^ The MMSE and WLM were applied to help fill out the CAM scale. The CAM was designed to be completed based on observations (not scores) made during brief but formal cognitive testing (with instruments such as the MMSE and WLM) requiring clinical judgment.^17^ Therefore, MMSE and WLM scores were calculated from patient worksheets after data collection. Proxies and family members, when available, were also interviewed regarding CAM elements to account for the fluctuating nature of delirium.

Demographic data, main diagnosis and comorbidities, length of ED stay, medications, history of neurological disorders, and drug use or abuse (including tobacco and alcohol) were examined in patient records. Education was recorded as years of education completed. The SOFA (Sequential Organ Failure Assessment) score^18^ and the Charlson comorbidity score^19^ were used to assess clinical severity and comorbidities. Medications were classified according to their potential association with delirium (opioids, benzodiazepines, tricyclic antidepressants, corticosteroids, H2-blockers, cardiac antiarrhythmic, and beta-blockers).^20^ Antipsychotics were evaluated separately.

Statistical analyses. Data analysis was performed using the Statistical Package for the Social Sciences (SPSS for Windows 18.0) software. Descriptive data (mean, SD and frequency) were calculated for demographic and clinical data. Parametric and non-parametric data were analyzed using Student's *t*-test or the Mann-Whitney test, respectively. Categorical variables were tested using the Chi-square test, with Yates correction or Fisher's exact test. Logistic regression models were constructed to evaluate the association between delirium and other variables. The first model included the MMSE, WLM, age and education, while the second model included the WLM, age and education, to determine the role of these tools in diagnosing delirium with the CAM. The third model included the clinical and demographic variables showing statistically significant association on the univariate analyses (main associated factors).

## RESULTS

During the 6-month period of the study, 435 patients were interviewed and 70 refused to participate or dropped out after signing the consent form. Therefore, the final sample comprised 365 participants. One hundred and twenty patients were evaluated in the morning, 126 in the afternoon, and 119 in the evening. Distribution among dayshifts according to the presence/absence of delirium was similar for both groups (p=0.849).

Clinical and demographic data are given in Table 1. Prevalence of delirium, according to the CAM, was 10.7%. Charlson comorbidity score did not differ between the two groups (p=0.454). Educational attainment differed significantly between delirium (mean education of 4.92 years) and non-delirium patients (mean education of 6.96 years) (Table 2). 

**Table 1:** Demographic and clinical data of total sample (N=365).

Variable	Distribution
Age*	58.05 ± 17.03
Sex (female)**	193 (52.9%)
Education (years)*	6.74 ± 3.94
Charlson comorbidity score	0.99 ± 1.71
CAM (with delirium)**	39 (10.7%)
MMSE*	21.02 ± 5.21

*mean ± SD; ** absolute and relative frequency.

**Table 2:**
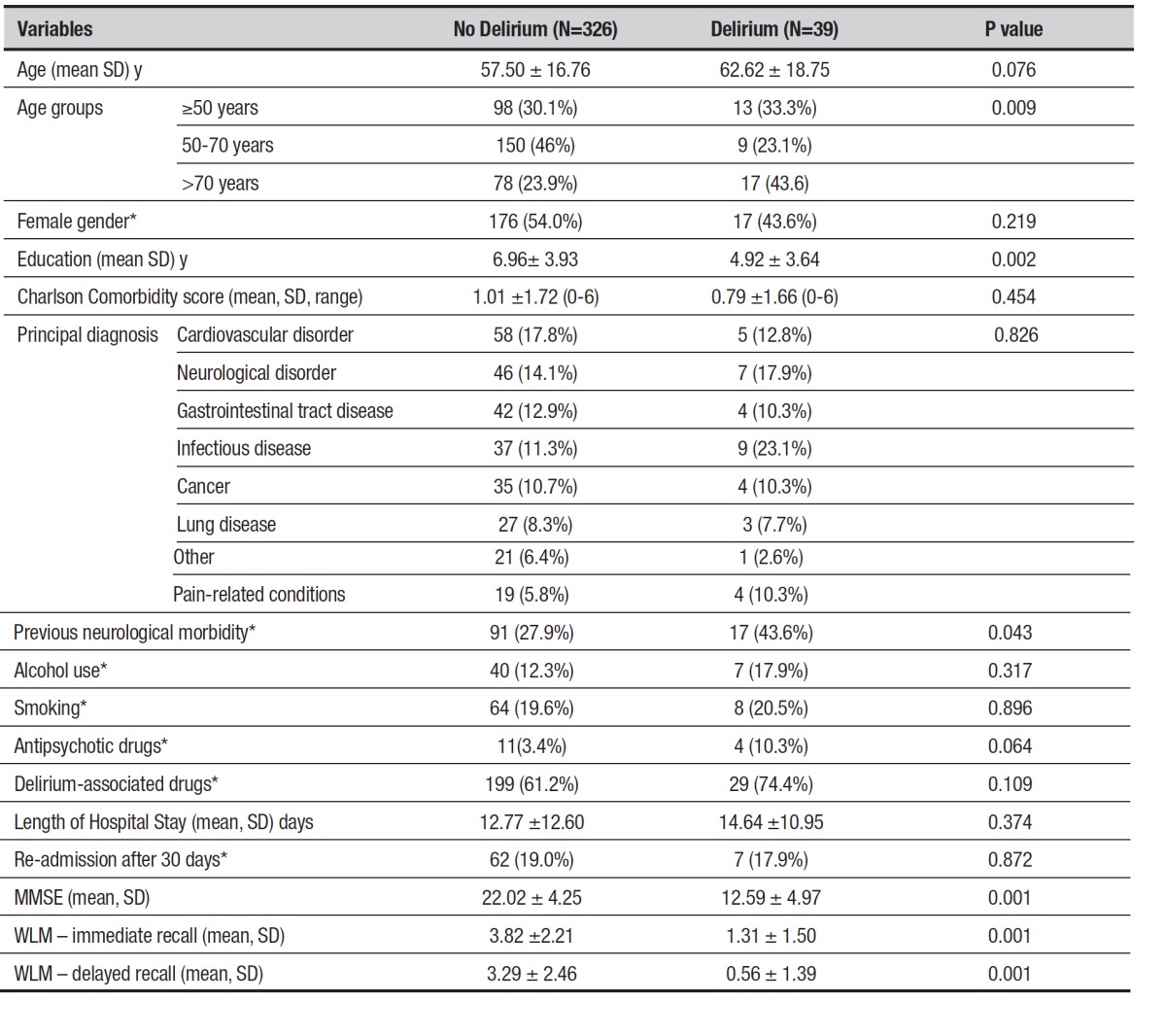
Demographic and clinical data according to presence/absence of delirium.

*Absolute and relative frequency; WLM: Wechsler Logical Memory.

Delirium patients had significantly lower MMSE scores than non-delirium patients (Table 2). Scores on the WLM (immediate and delayed recall) were also lower among patients with delirium. Both groups had significantly lower delayed scores than immediate recall scores (p=0.001). Occurrence of episodic memory impairment was 97.4% (N=38) among delirium patients and 76% (N=247) among non-delirium patients (c^2^=9.56; p=0.001). Age variability showed a tendency for significant difference between groups (Table 2). However, the distribution of patients after stratifying into age groups <50 years, 50-70 years, and ≥70 years showed a significant association with delirium (c^2^=9.50; p=0.008). More patients in the >70 years group presented delirium (N=17; 44%). 

Main diagnoses at ED admission were cardiovascular, neurological, gastrointestinal, oncologic and infectious disorders. The remaining conditions represented less than 20% of the diagnoses at emergency admission (genitourinary disease, pulmonary, rheumatologic, endocrinologic, psychiatric, and pain). Distribution of diagnoses showed significant association with age groups (c^2^=22.656; p=0.001). Younger delirium patients had more infectious disorders (67%) - especially HIV-related infections - than the other age groups. Older (>70 y) delirium patients had more oncologic and gastrointestinal diseases (70%).

Presence of previous neurological disorders was higher among delirium patients (c^2^= 4.11; p=0.043). Stroke (N=70; 19.2%) was the most frequent condition, followed by dementia and epilepsy.

Duration of hospital (ED) stay did not differ significantly between groups (p=0.374). 

Three logistic regression models were developed to evaluate the association of variables with delirium (independent of causal relationship since this was a cross-sectional investigation). For the first model, MMSE, WLM immediate and delayed scores, age and education were entered in the equation. Age (OR=0.97; 95%CI 0.94-1.00) and MMSE (OR=0.68; 95%CI 0.60-0.77) showed a significant association with delirium (Table 3).

**Table 3:** Variables in the three logistic models for the outcome "delirium".

Variables		B	Wald	P value	Odds ratio (OR)	95% C.I. for EXP (B)	
						Lower	Upper
Logistic Model 1	Education	0.096	1.727	0.189	1.101	0.954	1.270
	Age	-0.030	3.870	0.049	0.970	0.942	1.000
	MMSE	-0.391	38.385	0.000	0.676	0.597	0.765
	Logical memory Immediate	-0.118	0.479	0.489	0.889	0.637	1.241
	Logical memory Delayed	-0.262	1.751	0.186	0.770	0.522	1.134
Logistic Model 2	Education	-0.042	0.525	0.469	0.959	0.855	1.075
	Age	-0.014	1.320	0.251	0.986	0.963	1.010
	Logical memory Immediate	-0.299	4.216	0.040	0.742	0.558	0.986
	Logical memory Delayed	-0.483	7.901	0.005	0.617	0.441	0.864
Logistic Model 3	Education	-0.137	5.924	0.015	0.872	0.781	0.974
	Age	0.004	0.125	0.724	1.004	0.981	1.027
	Antipsychotic drugs	1.035	2.530	0.112	2.814	0.786	10.072
	History of neurological disease	0.438	1.513	0.219	1.562	0.767	3.181

For the second model, we removed MMSE was removed to determine episodic memory behavior in the delirium diagnosis without the strong association of the MMSE in ascertaining this diagnosis. Only the WLM (immediate and delayed scores) showed a significant association with the outcome (Table 3). 

For the third model, age, education, antipsychotic drugs, and history of neurological disorders were entered. Education was the only variable showing a significant association with delirium (OR =0.87; 95%CI 0.78-0.97; p=0.015) (Table 3).

## DISCUSSION

The prevalence of delirium in an ED of a large university hospital in southern Brazil was 10.7% using the CAM scale(Confusion Assessment Method).^12^ Patients with delirium exhibited lower education level; this association remained after controlling for other variables in a logistic regression model, supporting the role of education in the occurrence of delirium. In our study, 1 year of education was associated with a 1.15-fold decrease in the odds of delirium. This is very important since the average difference in education between groups was only 2 years and both groups had lower education averages as compared to developed regions of the world. Education, among other cognitive reserve indicators, is the most widely investigated. The cognitive reserve hypothesis postulates that there are individual differences in the ability to cope with brain pathology, such as AD-related plaques and tangles.^21^ The strong association of lower education with risk for dementia ranks education, according to some experts, as the most important protective factor for dementia.^21^ Education may increase brain reserve by promoting synaptic growth,^23^ and/or may foster cognitive reserve by generating new cognitive strategies.^24^ However, cognitive reserve has not been extensively studied in the context of delirium. A recent review on cognitive and brain reserve for many conditions affecting the central nervous system with a focus on delirium in two large cohorts of hospitalized older patients was conducted. Results revealed educational attainment as an important predictor of delirium, but a level of ≥3 years failed to show a significant association.^25^ In our study, average education was 5 years among those who developed delirium compared to 7 years among those who did not, i.e., each additional year represents an important source of protection.

The frequency of delirium was lower than expected considering the characteristics of the university hospital ED studied. At the facility, most severe and older patients from Porto Alegre city and Rio Grande do Sul state are treated through public funding and the number of patients usually exceeds the maximum capacity. Therefore, this noisy, sensory over-stimulated and sleep-disrupted setting was expected to contribute to a higher prevalence of delirium. On the other hand, the exclusion of critically ill and unstable patients - who are at higher risk of delirium - might explain the observed frequency (SOFA score >8 as exclusion criterion). This is corroborated by the low average Charlson comorbidity score observed in our sample (almost 1), indicating that patients included did not present severe comorbid conditions. The SOFA score, an assessment of organ dysfunction not specifically for sepsis, was primarily designed to describe morbidity and also evaluate mortality.^18^ This score was used to standardize assessment of clinical severity and exclude critically ill and unstable patients that would have prevented the application of the CAM.^5^ Furthermore, fluctuation of symptoms during the day or days (such as level of consciousness and inattention) could also contribute, at least in part, to the observed prevalence of delirium because patients were assessed only once.

Nevertheless, our findings are similar to those reported in the literature. Previous investigations in different settings using CAM for detection showed a range of prevalence values. In the study of Lewis et al., prevalence of delirium was 10%.^4^ Elie et al. reported a delirium prevalence of 9.6%.^5^ These investigations also took place in ED and found similar prevalence rates to our study. In a Brazilian study carried out in patients older than 18 years, the prevalence of delirium was 5.7%.^26^ Another investigation conducted in Brazil involving older patients (age >60 years) from hospital wards found a prevalence of 33%.^27^ In the study for the validation of the Brazilian CAM, prevalence of delirium was between 10 and 24% in hospitalized patients.^13^

Patients with delirium in our study had lower WLM test scores (immediate and delayed), suggesting poor episodic memory processing among these patients (and corroborating the classification of patients with CAM). This deficit is stronger than the association with education (as seen in the second regression model). However, since delirium is characterized by inattention, poor episodic memory performance can be expected. The use of the test in helping to fill out the CAM was also important for the association.

We observed a noteworthy association of age group with delirium, since patients with a wide age range (20-94 years) were included. The highest frequency of delirium was shown by the age group >70 years (44%) followed by the group <50 years (33%) characterizing a bimodal distribution. Younger patients also had a higher frequency of infectious disorders, especially HIV-related, contributing to the occurrence of delirium. Older patients presented more oncologic and gastrointestinal disorders which, besides age, contributed to the occurrence of delirium. Age is a widely accepted risk factor for delirium. Additionally, the frequency of episodic memory deficits was higher among delirium patients, suggesting at least a degree of inattention and potential influence of the ED milieu (or nature of illness). 

Finally, studying the distribution of delirium in different populations is of importance because delirium is a common and often under-recognized condition. Delirium is also an interesting model of acute and transient cognitive impairment for understanding the role of cognitive reserve. To the best of our knowledge, this is the first study estimating the prevalence of delirium and its association with educational attainment (cognitive reserve) in a Brazilian emergency department. However, further investigations clarifying the causative relationship of different cognitive reserve estimates in this population are warranted.
